# Cancer of Cervix in Nullipara

**Published:** 1915-06

**Authors:** 


**Cancer of Cervix in Nullipara,** aged 17, with removal of cauliflower growth and apparent cure, is reported by Ayres in Am. Jour. of Obs.

